# Mimological Reveries? Disconfirming the Hypothesis of Phono-Emotional Iconicity in Poetry

**DOI:** 10.3389/fpsyg.2016.01779

**Published:** 2016-11-15

**Authors:** Maria Kraxenberger, Winfried Menninghaus

**Affiliations:** Language and Literature, Max Planck Institute for Empirical AestheticsFrankfurt, Germany

**Keywords:** poetry, phonological iconicity, joy, sadness, tonal contrasts, frequencies of occurrence of phonemes

## Abstract

The present study retested previously reported empirical evidence suggesting an iconic relation between sound and emotional meaning in poetry. To this end, we analyzed the frequency of certain phoneme classes in 48 German poems and correlated them with ratings for emotional classification. Our analyses provide evidence for a link between the emotional classification of poems (joyful vs. sad) and the perception of tonal contrast as reflected in the attribution of phenomenological sound qualia (bright vs. dark). However, we could not confirm any of the previous hypotheses and findings regarding either a connection between the frequencies of occurrence of specific vowel classes and the perception of tonal contrast, or a relation between the frequencies of occurrence of consonant classes and emotional classification.

## Introduction

A potentially non-arbitrary, “natural” (gr. *physei*), or “iconic” relation between sound and meaning in language has been a controversial topic since Greek antiquity (Plato, 1892; for a detailed historical overview, see Genette, 1995; on the principle of the arbitrariness of signs, see De Saussure, 1916/1983). Recent (psycho-)linguistic studies have suggested that phonological iconicity is a property of languages that should be acknowledged as an important addition to the principle of the arbitrariness of the linguistic sign (Perniss et al., 2010; Myers-Schulz et al., 2013; Perniss and Vigliocco, 2014; for an overview see Hinton et al., 2006; Schmidtke et al., 2014). In particular, poetry has often served as a testing ground for the hypothesis of an “inmost, natural similarity association between sound and meaning” (Jakobson and Waugh, 1979/2002, p. 182; see also Valery, 1958; Jakobson, 1960; Fónagy, 1961; Tsur, 1992; Whissell, 2002, 2011; Pope, 2010; Schrott and Jacobs, 2011; Aryani et al., 2016). Specifically, two studies by Albers (2008) and Auracher et al. (2010) provided empirical support for the hypothesis of phono-emotional iconicity in poetry. We (re-) tested the findings of these studies on a corpus of poems that is far more varied in authorship and stylistic features than were the corpora of the original studies.

### Joy and sadness

Just as topical understandings of poetry place a strong emphasis on the role of sound, poetry has also frequently been associated with expressing and eliciting emotions (Hegel, 1986; Winko, 2003; Meyer-Sickendiek, 2011; Lüdtke et al., 2014). Following other empirical studies on phonological iconicity in poetry, we too focused on the basic emotions of joy and sadness (Russell, 1980; Ekman, 1992; Jack et al., 2014). Phenomenological accounts of emotional qualities have conceived of joy, happiness, and pleasure as being mainly characterized by ease, uplift, and spatiotemporal expansion (German: *Weitung*), i.e., by a person's feeling of being light, free, and flowing (Schmitz, 1969; Demmerling and Landweer, 2007). Sadness, on the other hand, is typically characterized by the opposite features: as bleak, compressed, heavy, and downward-oriented, as a feeling of oppression and depression (Schmitz, 1969), and as anxious, passive, and burdened (Demmerling and Landweer, 2007).

Moreover, positive emotions are often linked to brightness, while negative emotions are associated with darkness (cf. Schmitz, 1969; Demmerling and Landweer, 2007; Albers, 2008).

These descriptions were confirmed by two empirical studies (Boyatzis and Varghese, 1994; Hemphill, 1996)^1^.

### Felt and perceived emotions

Psychological theories of emotions conceive of prototypical emotions as processes comprising different emotion components: cognitive and non-cognitive appraisals (novelty, intrinsic pleasantness, relevance, attributions of agency, coping potential, conduciveness for our goals/needs, etc.), peripheral-physiological processes, a subjective feeling component, motor expression patterns, action tendencies, memory and attentional processes (Frijda, 1986; Clore et al., 1987; Russell and Barrett, 1999; Russell, 2003; Scherer, 2005). Emotions have been mapped onto the multi-dimensional affect space, with the three largely agreed upon dimensions defined by Wundt as valence (positive vs. negative), activation/arousal, and potency (Wundt, 1896; Schlosberg, 1954; Fontaine et al., 2007; Veirman and Fontaine, 2015).

In the context of the present study, several aspects of emotion processing are of importance. The first is explicit emotional classification, i.e., assigning the appropriate emotion term to the poems' key emotional tonality. A classification of this type is likely to be primarily driven by perceived, or decoded, emotional content. Such decoding does not necessarily require the readers of the poems to actually feel joyful or sad themselves. However, we were precisely interested in non-semantic, psychoacoustic dimensions of how readers perceptually sense, or intuitively feel, a poem's emotional tonality. After all, this is what the hypothesis of phono-emotional iconicity is about. Specifically, we tested whether or not we can confirm the results of Auracher et al. (2010) regarding a perceptual sound-emotion-link in poetry.

### Front vs. back vowels and the perception of tonal contrast

Research on phonological iconicity has repeatedly assumed a link between the perception of tonal contrast (i.e., perceiving something as rather bright/light or dark) and vowel quality for an array of different languages. As early as 1876, Gustav Theodor Fechner, the founding figure of empirical aesthetics, suggested that, in general, “a, e, i appear as brighter and o, u as darker” (Fechner, 1876, p. 318, our translation)^2^. Similar hypotheses were advanced in more recent studies (Jakobson and Waugh, 1979/2002; Tsur, 1992, 1997; Wrembel, 2009; Moos et al., 2014).

Fechner's grouping of vowels is in line with present-day distinctions between front and back vowels, except for the case of the centralized /a/. The distinction between front and back vowels is based on articulation and hence on the physiology of the human vocal tract. Generally, vowels and vowel quality are distinguished in a vertical and a horizontal dimension, and are positioned in the space of two different resonance frequencies (formants). Formants, main acoustic features of vowel quality, are peaks of the sound spectrum, i.e., accumulations of acoustic energy at certain frequencies (Moos et al., 2014). Formant 1 (F1, vertical dimension) correlates with the oral cavity's degree of opening (closed to open) and formant 2 (F2, horizontal dimension) with a fronting or backwards movement of the tongue body. This leads to a distinction between front (for German, e.g. /i/ or /e/), back (for German, e.g., /u/ or /o/), and centralized positions (for German: /a/). The distinction between front and back vowels differs depending on linguistic approaches and language-specific characteristics (see “Procedure for the Phonological Analyses”).

To our knowledge, apart from the analysis of single utterances or single poems (e.g., Tsur, 1992, 1997)^3^, research on phonological iconicity has not yet empirically tested the hypothetical link between front/back vowels and the perception of tonal contrast across a larger number of poems.

### Plosives and nasals in joyful and sad poems

Several empirical studies have claimed evidence for a relation between the frequencies of occurrence of consonants and the emotional classification of poems (joyful vs. sad) across different languages and language families. Most of these studies used the physiology of articulation as the basis for attributing emotional meaning to certain phonemes or phoneme classes; they consequently focused on phonemic contrasts (for a short overview, see Miall, 2001). Thus, a study by Albers (2008) reported different frequencies of occurrence of plosives and nasals in joyful and sad poems. Albers's study is based on findings from a survey study involving German and Brazilian participants (Wiseman and Van Peer, 2003). This survey indicated that the use of certain plosives was perceived to be more appropriate in a pleasant context (for instance, a wedding), whereas the use of the nasals /m/ and /n/ was reportedly more suitable in sad contexts (such as funerals). In line with these findings, Albers (2008) reported that the plosives /p/, /b/, /t/, and /d/ occur most frequently in a corpus of Old Egyptian hymns as well as in a selection of hymns by the German poet J. W. von Goethe. By contrast, the nasals /m/ and /n/ were more frequent in Old Egyptian lamentations and ballads by Goethe. A related study drawing on corpora of German, Chinese, Russian, and Ukrainian poems showed that, for each language, the poem with the highest frequency of the plosives /p/, /b/, /t/, and /d/ was rated by native participants as joyful and high in activation whereas, again for each of these languages, the poem with the highest frequency of nasals (/m/, /n/) was evaluated as sad and low in activation (Auracher et al., 2010). These three studies suffer, however, from substantial limitations: they neither included the entire group of plosives (/p, b, t, d, k, g/, see, e.g., Wiese, 1996; Kohler, 1999; Kuzla and Ernestus, 2011) nor the entire class of nasals of the German language (/m, n, ŋ/; see, e.g., Wiese, 1996; Kohler, 1999). Specifically, they did not consider /k/ and /g/ in their analyses of the class of plosives, while /ŋ/ was disregarded regarding the class of nasals. Moreover, the study by Auracher et al. (2010) collected ratings exclusively for the two individual poems in each language that featured the highest frequencies of plosive and nasal sounds, but not for all poems. As a result, it is not clear whether these relational frequencies can actually predict the emotional classification of all poems in the corpus—and consequently, whether they can in fact be understood as group-differentiating variables. Furthermore, the results of the three studies differ from those of previous research: Fónagy (1961) found /t/ to be more frequent in aggressive and hence negatively valenced poems, and Whissell (1999) reported that the plosives /d/, /b/, and /t/ tend to be more dominant in unpleasant words and to correlate negatively with pleasantness. Additionally, Miall (2001) found higher frequencies of occurrence of plosives in poetic verses that were interpreted as expressing negative experiences^4^. Given this divergence of hypotheses and findings, we reasoned that a replication and extension of Auracher's approach—one that circumvents its limitations—might provide more clarity.

## Methods

### Corpus

We compiled a corpus of 24 joyful^5^ and 24 sad German poems. We based this qualitative a priori classification on the poems' emotional content and phenomenological descriptions of emotional quality (Schmitz, 1969; Demmerling and Landweer, 2007; see above).

Selected poems were written, or first published, between 1828 and 1978 and ranged from 4 to 24 verses (*M* = 13.60; *SD* = 4.58). We included the titles in our phonological analyses and also presented them in the survey study (for the importance of titles, see Moretti, 2013). The 48 poems were written by 39 authors; two authors were represented with three poems each, and five authors with two poems each (for a list of authors and titles, see Table 1). Thirty-one of the poems feature a clear and consistent meter, while 17 poems are not metered in any narrower sense. Meter was measured using Metricalizer (Bobenhausen, 2011) as a first orientation; mistakes were manually corrected. Forty-one of the poems feature end rhymes. Thus, the selected poems include a considerable variation in authorship, time of origin, length and form.

**Table 1:** **Titles, authors, publication date, general features, and mean-emotion ratings of the analyzed poems and percentage of participants that were familiar with the respective poem**.

**Title**	**Author**	**Publication date**	**No of lines**	**End-rhymed**	**Consistent meter**	**Joyful vs. Sad**	**Emotion-Rating**	**Familiarity**
*Tristesse*	Benn, Gottfried	1956	16	Yes	Yes	Sad	5.69	6.3%
*Sommersonett*	Bergengruen, Werner	1950	14	Yes	Yes	Joy	1.94	0
*Novemberabend*	Boldt, Paul	1912	8	Yes	Yes	Sad	5.38	0
*Der Kuss*	Borchert, Wolfgang	1946	12	Yes	Yes	Joy	3.38	0
*Doppelte Freude*	Busch, Wilhelm	1909	8	Yes	Yes	Joy	2.38	6.3%
*Rückkehr*	Cordan (Horn), Wolfgang	1951	12	Yes	No	Sad	6.38	0
*Blick ins Licht*	Dehmel, Richard	1913	21	Yes	Yes	Joy	3.56	0
*Fähre Schenkenschanz*	Delius, Friedrich Christian	1981	8	Yes	No	Joy	3.69	0
*Sterben*	Ehrenstein, Albert	1961	13	No	No	Sad	5.50	0
*Heimkehr*	Ehrenstein, Albert	1961	12	Yes	No	Sad	5.81	0
*Call it love*	Enzensberger, Hans Magnus	1957	16	No	No	Joy	3.06	0
*april*	Enzensberger, Hans Magnus	1963	23	No	No	Sad	2.56	0
*trennung*	Enzensberger, Hans Magnus	1957	18	No	No	Joy	5.88	6.3%
*Freundliche Nähe*	Ernst, Otto	1917	16	Yes	Yes	Joy	2.00	0
*Schön und gut und klar und wahr*	Gernhardt, Robert	1990	12	Yes	No	Joy	3.25	0
*Trauermarsch*	Goll, Yvan	1960	13	Yes	Yes	Sad	6.69	6.3%
*O leuchtender Septembertag*	Haller, Paul	1922	12	Yes	Yes	Joy	2.38	6.3%
*Spät*	Hardekopf, Ferdinand	1963	12	Yes	Yes	Sad	6.31	0
*Regen*	Hatzfeld, Adolf von	1919	12	Yes	Yes	Sad	5.63	0
*Schwermut*	Henckell, Karl	1921	24	Yes	Yes	Sad	6.25	0
*Im Nebel*	Hesse, Hermann	1905	16	Yes	Yes	Sad	6.19	6.3%
*Fröhlichkeit*	Heym, Georg	1911	12	Yes	Yes	Joy	2.25	0
*Letzte Wache*	Heym, Georg	1964	16	Yes	Yes	Sad	6.88	0
*Nicht alle Schmerzen*	Huch, Ricarda	1971	12	Yes	No	Sad	5.75	6.3%
*Das berühmte Gefühl*	Kaléko, Mascha	1978	14	Yes	Yes	Sad	5.38	0
*Traurigkeit*	Kalkowska, Eleonore	1916	10	Yes	No	Sad	6.00	0
*Das Glück im Spiel*	Klabund	1927	14	Yes	Yes	Joy	3.50	0
*Liebeslied: Dein Mund*	Klabund	1927	16	Yes	Yes	Joy	2.63	6.3%
*Erfüllung*	Klemm, Wilhelm	1919	12	Yes	No	Joy	1.89	6.3%
*Freude*	Krzyzanowski, Otfried	1919	4	No	No	Joy	2.50	0
*Dämmerung*	Lasker-Schüler, Else	1943	10	Yes	Yes	Sad	5.50	0
*Liebeslied*	Lichtenstein, Alfred	1919	6	No	No	Joy	1.94	0
*Der Rauch auf dem Felde*	Lichtenstein, Alfred	1914	25	No	Yes	Sad	6.13	0
*Nachtmusik*	Loerke, Oskar	1958	12	Yes	No	Sad	5.38	0
*Radfahrt*	Malkowski, Rainer	1977	14	Yes	No	Joy	2.63	0
*Licht ist Liebe*	Morgenstern, Christian	1914	12	Yes	Yes	Joy	3.44	0
*Das ästhetische Wiesel*	Morgenstern, Christian	1905	11	Yes	No	Joy	2.50	25%
*Die Windhosen*	Morgenstern, Christian	1910	12	Yes	Yes	Joy	3.63	0
*Er ist's*	Mörike, Eduard	1828	10	Yes	Yes	Joy	1.50	62.5%
*Vereinsamt*	Nietzsche; Friedrich	1882	23	Yes	Yes	Sad	6.06	0
*Das Leben ist gut und licht*	Rilke; Rainer Maria	1913	8	Yes	Yes	Joy	2.44	0
*Morgenwonne*	Ringelnatz, Joachim	1933	12	Yes	Yes	Joy	1.63	0
*Nach derTrennung: Lichterfelde*	Ringelnatz, Joachim	1929	20	Yes	Yes	Sad	5.38	0
*Elegie*	Schwachhofer, René	1964	13	Yes	No	Sad	6.63	0
*Pans Trauer*	Stadler, Ernst	1911	14	Yes	Yes	Sad	5.25	0
*Das Licht*	Strub, Urs Martin	1946	9	Yes	Yes	Joy	3.19	0
*Die Zerwartung*	Thoor, Jesse	1965	14	Yes	Yes	Sad	6.13	0
*Ostersamstag*	Wagner, Christian	1890	20	Yes	Yes	Sad	6.25	6.3%

We opted for a more contemporary corpus, because previous empirical research on phono-emotional iconicity has largely refrained from using contemporary poems (see Schmidtke et al., 2014, for a review; for an analysis of poems from the twentieth century, see Aryani et al., 2016). Consequently, all poems, except E. Mörike's *Er ist's* (1829) and F. Nietzsche's *Vereinsamt* (1882), were written in the twentieth century.

In order to minimize familiarity effects (Zajonc, 1968; Zajonc and Rajecki, 1969; North and Hargreaves, 1995; Obermeier et al., 2013), we selected poems that we expected to be relatively unknown to our participants, and also asked them whether they knew the poems they were presented with (see below).

### Procedure for the phonological analyses

As a first step, we executed a grapheme-to-phoneme conversion for all poems using WebMAUS (Reichel, 2012; Reichel and Kisler, 2014) and counted the number of occurrences of each phoneme within each poem. Because the phonemization of Modern Standard German includes several problematic cases (for a review, see Wiese, 1996), all diphthongs and affricates were counted as both monophonemic and biphonemic units. We considered both classifications in our analyses. Since the results were the same, unless otherwise specified, we report only the classification that treated diphthongs and affricates as monophonemic units. Glottal stops were not considered, since there is agreement that they “should not be treated as a phoneme” (Wiese, 1996, p. 16).

To make sure that the phonological material included in our corpus matched a common phonological distribution in poetry, we calculated the percentages (relative frequencies) of all phonemes across all poems in our corpus and compared them with those calculated by Meier (1964) for a different poetry corpus. Because Meier's classification of phonemes lacks phonological accuracy (e.g., der [d e: a] is used as an example for /r/; Meier, 1964, p. 253), the comparison was subject to a few limitations. In order to avoid problematic phoneme groupings, we only included the consonants /b, d, f, g, h, k, l, m, n, ŋ, p, t, s, v, x, z, ʃ, ç/ in the comparison of Meier's corpus and ours^6^. In the case of vowels, we analyzed /i:, ɪ, e:, ɛ, ɛ:, o:, ɔ, u:, ʊ, a, a:/.

The difference between the relative frequency of consonants in our corpus and in Meier's range from −0.72 (for /g/) to 1.97% (for /t/), and the range for vowels varies from −0.37 (for /i/) to 0.07% (for /e/).^7^ The ranking order for the vowels is identical in both corpora. The consonant that varies most between the two corpora, /t/, is the second most frequent consonant in our corpus as well as in Meier's (1964). Thus, the frequencies of occurrences of phonemes in our corpus do not essentially differ from those in Meier's corpus.

#### Normalized frequencies of phoneme occurrences

We calculated *normalized frequencies of occurrence* for all phonemes by dividing the number of occurrences of each phoneme in a poem by the sum of all phonemes in the poem. To calculate the normalized frequencies for an entire class of phonemes (front and back vowels, nasals, and plosives), we added up all normalized frequencies for the constituent phonemes. This approach also allowed for comparisons between individual poems (regardless of their differences in absolute length), between relational phoneme classes, between multiple (related) classes, and also between single phonemes without a need to determine phonological relations a priori or to use non-poetic corpora (e.g., rated word lists) for comparison (cf., Whissell, 2000).

We followed the classification of vowels given in Wiese's feature matrix (1996), which categorizes /i:, ɪ, e:, ɛ, ɛ:, y:, ʏ, ø:, œ/ as front vowels and /o:, ɔ, u:, ʊ/ as back vowels. However, taking other classifications of front vs. back vowels likewise into account, we also compared /i, e/ vs. /u, o/ (Jakobson, 1962) and /i/ vs. /u/ (Tsur, 1992)^8^.

#### Relational frequencies of phoneme occurrences

In order to replicate the results of Auracher et al. (2010), we applied the same analyses to the plosives /p/, /b/, /t/, and /d/ and the nasals /m/ and /n/. That is, we counted the respective occurrences of these phonemes and calculated *relational frequencies of occurrence* in terms of a plosive/nasal ratio, based on the absolute frequencies of occurrence in each poem. This allowed us to examine relational phoneme classes. In contrast to the use of normalized frequencies of occurrence, this approach does not allow for a comparison with other phoneme classes (see above). We also calculated the relative frequencies of occurrence in terms of the ratio of nasals/plosives as well as the relational frequencies for all German consonants that can be assigned to the classes of plosives and nasals.

### Participants

One hundred and twenty-eight participants (84 women, 44 men) took part in the rating study. The mean age was 24.5 years (*SD* = 4.36, min = 18, max = 37). Inclusion criteria for study participation were German as native language and full legal age. Four of the participants (3.1%) had been brought up bilingually, with German being one of their mother tongues. All experimental procedures were undertaken with informed consent of each participant.

### Questionnaire

The questionnaire included two unipolar rating items for how positive (hereafter: Positivity) and negative (hereafter: Negativity) participants perceived the content of the poems to be; the items ranged from 1 (not at all) to 7 (extremely). Another item (hereafter: Emotion) was used to measure whether participants assigned the perceived emotional tonality of the respective poem rather to the pole of joy (1) or to that of sadness (7). Using the question How does the poem sound? (Sound), we collected ratings of perceived tonal contrast ranging from 1 (bright) to 7 (dark)^9^.

The sequence of the items within each set of questions was randomized between participants. Participants were also asked to indicate whether they knew the respective poems (hereafter: Familiarity). Finally, participants reported their age (in years), gender (female or male), and affinity (hereafter: Affinity) for poetry, the latter by stating to what extent they generally enjoy reading or listening to poetry on an item ranging from 1 (not at all) to 7 (very much).

### Procedure for the rating study

Participants were instructed to silently read each poem twice in a calm and attentive manner. This instruction was used because previous studies employing a rereading paradigm suggest that the effects of literary language consolidate over time and that repeated reading supports a greater “depth of appreciation” (Dixon et al., 1993, p. 17; cf. also Hakemulder, 2004). To increase participants' attention to the poems' sound patterns, they were instructed upon second silent reading to read the poem as if they were reading it aloud.

Given the size of the corpus, we opted for a between participants design. To reduce possible fatigue and carryover effects, we presented only a few stimuli per participant. The 48 poems were divided into 8 groups of 6 poems each. As a result, each poem received 16 ratings, and each participant rated 6 poems—three joyful and three sad ones in a randomized order.

### Statistical analysis

All analyses, apart from the linear mixed effects analyses reported below, were conducted in SPSS (IBM SPSS Statistics for Windows, Version 22.0, IBM Corp., 2013). A visual inspection of normal Q-Q plots showed that both our behavioral and phonological data were approximately normally distributed. We used R (R Core Team, 2013) and lme4 (Bates et al., 2014) to perform linear mixed effects analyses. *P*-values were obtained by likelihood ratio tests of the full model with the tested effect against the model without this effect. Apart from the linear mixed effect analyses, our analyses are—if not otherwise indicated—based on mean values.

## Results

### Familiarity and affinity

To control for possible effects of participants' familiarity with the poems, we excluded two joyful poems from further analyses because they were familiar to more than 10% of the participants (for an overview of all poems, see Table 1): *Das ästhetische Wiesel* by Ch. Morgenstern, known to 4 of its 16 raters (25%), and *Er ist's* by E. Mörike, known to 10 of its 16 raters (62.5%).

On average, participants indicated an affinity of 5.05 for reading or listening to poetry (*SD* = 1.58, min = 1, max = 7). We performed a linear mixed effects model for the perception of the poems' Emotion as dependent variable, and Affinity as predictor variable, including random intercepts for participants and poems, as well as by-participant and by-poem random slopes. The analysis of the relationship between ratings of Emotion and participants' affinity for poetry showed no significant result [$\chi_{\left(1\right)}^{2}$ = 1.91; *p* = 0.17; ß = −0.05; *SE* = 0.03; *t* = 1.4].

### Emotional classification of the poems

As a first step, we examined whether or not the participants confirmed our preclassification of the poems as either joyful or sad. To this end, we inspected the mean values of all poems on the item Emotion. The means of the poems that were preclassified as joyful (*M* = 2.72, *SD* = 0.65, min = 1.63, max = 3.69) were all below the midpoint of the scale (4), whereas the means of the poems that were preclassified as sad (*M* = 5.93, *SD* = 0.46, min = 5.25, max = 6.88) were all above the midpoint (also, see Table 1 for mean ratings for all poems on the Emotion-item).

This result was corroborated by highly significant correlations between our preclassification of the poems as either joyful or sad (coded as 0 and 1, respectively) and participants' ratings for Emotion, Positivity and Negativity (all | *r* | = 0.92; *p* < 0.001). The result was further supported by a linear mixed effects analysis with Emotion as dependent variable and preclassification as independent variable with random effects for participants and poems [$\chi_{\left(1\right)}^{2}$ = 70.71; *p* ≤ 0.0001; ß = −3.04; SE = 0.23; *t* = −13).

As univariate analyses of variance (ANOVAs) showed, participants rated the content of the joyful poems as more positive (*N* = 22, *M* = 5.20, *SD* = 0.69) than the content of the sad poems [*N* = 24, *M* = 2.22, *SD* = 0.60, *F*_(1, 45)_ = 245.58, *p* < 0.001, ŋ^2^_*p*_ = 0.85]. Inversely, the content of the sad poems was rated as significantly more negative (*M* = 5.42, *SD* = 0.54) than the content of the joyful poems [*M* = 2.43, SD = 0.70, *F*_(1, 45)_ = 246.04, *p* < 0.001, ŋ^2^_*p*_ = 0.86].

### Phenomenological perceptions of tonal contrast

**Figure 1:**
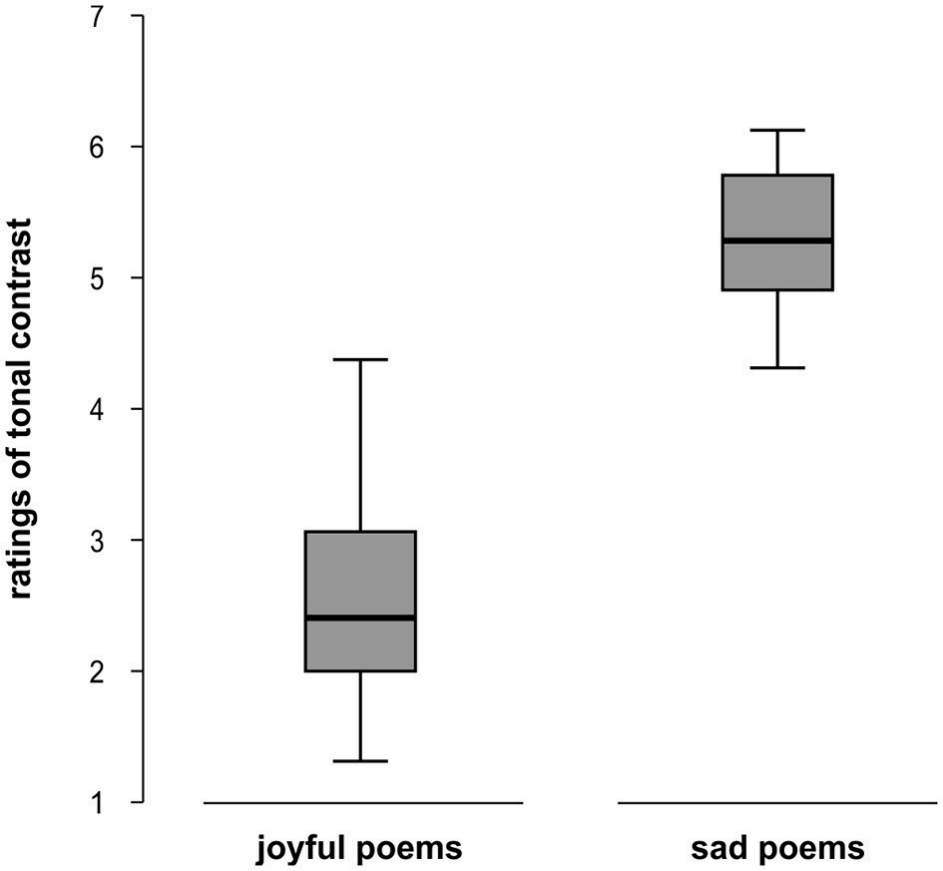
**Boxplots showing participants' ratings of tonal contrast for joyful and sad poems on a 7-point scale (1: bright; 7: dark)**.

An ANOVA revealed that phenomenological perceptions of tonal contrast as measured by the Sound qualia “bright” and “dark” differed significantly between the two groups of poems [*F*_(1, 45)_ = 184.45, *p* < 0.001, ŋ^2^_*p*_ = 0.81], with joyful poems perceived as sounding brighter (*M* = 2.69, *SD* = 0.76) and sad poems as sounding darker (*M* = 5.29, *SD* = 0.53; see Figure 1).

### Front and back vowels and the perception of tonal contrast

**Figure 2:**
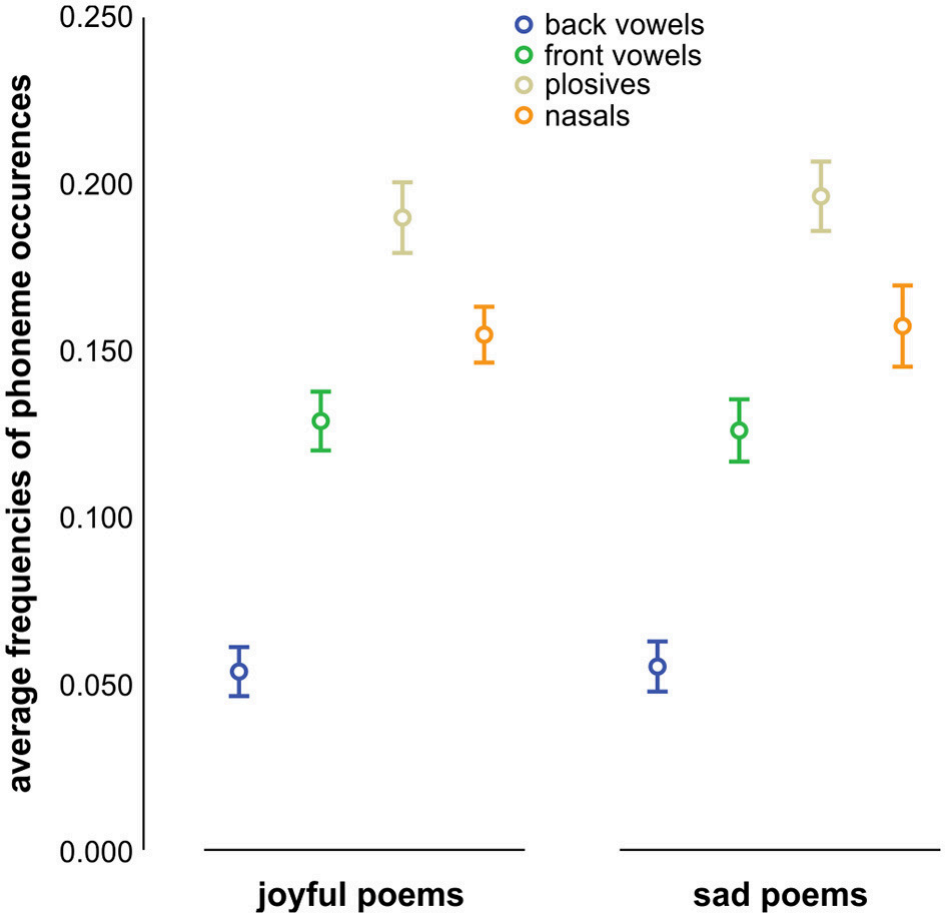
**Means and standard errors of the mean (SEM) of frequencies of phoneme occurrences for joyful and sad poems**. Colors indicate the respective phoneme classes.

To test the hypothesis that the perception of brightness or darkness is related to the normalized frequencies of front and back vowels, we performed three linear mixed effects analyses (one for each definition of vowel class i.e., front vs. back vowels as defined by Wiese (1996), /i/ vs. /u/ Tsur, 1992, and /i, e/ vs. /u, o/ Jakobson, 1962). In doing so, we regressed participants' perception of Sound on the frequencies of front and back vowels, including intercepts for participants and poems as random effects. These analyses showed no significant effects of the frequencies of front and back vowels on the perception of Sound [all $\chi_{\left(2\right)}^{2}$ ≤ 1.48; all *p* ≥ 0.5; all ß(back vowels) ≤ 6.64 all *SE* ≥ 13.66; *t* ≤ 0.49; all ß(front vowels) ≤ 14.68.64 all *SE* ≥ 11.97; *t* ≤ 1.23]. Moreover, front and back vowels were almost equally distributed between joyful and sad poems (cf. Figure 2).

### Plosives and nasals in joyful and sad poems

In order to examine whether the joyful and sad poems differ in terms of frequencies of occurrence of plosives and nasals, we conducted two ANOVAs for each of the classifications of plosives and nasals (a) as given by Albers (2008) and Auracher et al. (2010), and (b) including all plosives and nasals, respectively (cf. Table 2). These analyses of variance were performed using the relational frequencies of plosives by nasals and of nasals by plosives, respectively, as dependent variable. We also applied ANOVAs to the normalized frequencies of plosives and nasals (one excluding and the other including /k/, /g/, and /ŋ/). None of the results showed any significant differences between joyful and sad poems [all *F*_(1, 45)_ ≤ 1.93, all *p* ≥ 0.17]. Consequently, we did not find higher mean values for the frequencies of plosives in joyful poems or for the frequencies of nasals in sad poems (cf. Table 2, as well as well as Figure 2).

**Table 2:** **Descriptive statistics and results of analyses of variance of joyful and sad poems with regard to the frequencies of occurrence of plosives and nasals**.

	***M (n)***	***SD***	***F***	***P***	**ŋ^2^_*p*_**
RF Plosives (Auracher et al., 2010)	(a) 1.04 (22) (b) 1.12 (24)	(a) 0.24 (b) 0.32	1.05	0.31	0.02
RF Nasals (Auracher et al., 2010)	(a) 1.02 (22) (b) 0.96 (24)	(a) 0.24 (b) 0.28	0.49	0.49	0.01
RF Plosives (all)	(a) 1.02 (22) (b) 0.96 (24)	(a) 0.25 (b) 0.34	0.29	0.60	0.01
RF Nasals (all)	(a) 1.25 (22) (b) 1.30 (24)	(a) 0.17 (b) 0.21	0.05	0.84	0.001
NF Plosives (Auracher et al., 2010)	(a) 0.83 (22) (b) 0.82 (24)	(a) 0.03 (b) 0.02	1.93	0.17	0.04
NF Nasals (Auracher et al., 2010)	(a) 0.15 (22) (b) 0.16 (24)	(a) 0.02 (b) 0.03	0.04	0.84	0.001
NF Plosives (all)	(a) 0.19 (22) (b) 0.20 (24)	(a) 0.02 (b) 0.03	0.80	0.38	0.02
NF Nasals (all)	(a) 0.16 (22) (b) 0.20 (24)	(a) 0.01 (b) 0.01	0.13	0.73	0.003

*Note: Means are given for (a) joyful and (b) sad poems. RF stands for relational frequencies of occurrence, and NF for normalized frequencies of occurrence*.

Our two measures of relational frequencies of plosives and nasals were highly correlated (Pearson Correlation, two-tailed, *N* = 48, *r* = −0.96, *p* ≤ 0.001). To test whether the poem with the highest relational frequency of plosives tends to be perceived as joyful and the poem with the highest relational frequency of nasal phonemes as sad, we produced two ranked lists—one ordering the poems by their relational frequency of plosives and the other by their relational frequency of nasal sounds. The poem with the highest relational frequency of plosive sounds (1.82) was Herdekopf's *Spät* (1963), and the poem with the highest relational frequency of nasal sounds (1.57) was Loerke's *Nachtmusik* (1958). As the rating for Emotion showed, participants classified both poems as sad (*M*_Spä*t*_ = 6.31, *SD*_Spä*t*_ = 0.87; *M*_Nachtmusik_ = 6.25, *SD*_Nachtmusik_ = 0.96), thus highlighting the above-reported result that the relational frequency of nasal vs. plosive phonemes does not predict the perception of emotional tonality.

Similarly, a linear mixed effects analyses regressing Emotion on the frequencies of plosives and nasals, including intercepts for participants and poems as random effects, did not show any significant effect of the phonological variables on the perceived emotional tonality [all $\chi_{\left(2\right)}^{2}$ ≤ 1.86; all *p* ≥ 0.4].

## Discussion and outlook

Our results provide evidence for a link between the emotional classification of poems and the phenomenological perception of bright vs. dark sound qualia. However, we found no differences between joyful and sad poems with regard to the frequencies of occurrence of front and back vowels that might underlie these phenomenological perceptions. Thus, our study does not confirm the hypothesis of a non-arbitrary link between particular phoneme inventories and emotion perception in poetry reading.

The poem with the highest relational frequency of plosives was rated as sad and not, as would be expected based on previous findings, as joyful. At the same time, the poem with the highest frequency of nasals was also rated as sad. Thus, the results of Auracher et al. (2010) could not be replicated. Furthermore, joyful poems did not differ from sad poems in terms of relational or normalized frequencies of occurrence of plosives and nasals. Consequently, an iconic relation between these phoneme classes and emotional classification could not be confirmed.

The discrepancy between our results and those of Auracher et al. (2010) and Albers (2008) could be due to differences of the corpora used: The anthology Auracher et al. drew upon is specifically directed at students in their third or fourth year of high school (cf. Bruns, 1921). Only 14 authors, with up to 19 poems per author, wrote the 138 poems included in the anthology; this strongly limits the results in terms of representative value. J. W. von Goethe, for instance, was represented with 17 poems. Moreover, three of these poems by Goethe as well as an earlier version of one of these poems (i.e., 23.5%) were already included in the corpus used by Albers (2008), which comprises only 13 poems altogether^10^. This overlap may have contributed to the converging results reported by these two studies. In contrast, our corpus was designed not to have any overlap with those used in the preceding studies. Results show that previous findings cannot be generalized beyond the corpora used in the respective studies.

A parsimonious explanation of our results could be that the attribution of a bright vs. dark sound impression for joyful vs. sad poems is an effect of supra-segmental parameters—specifically, vocal emotional expression—rather than of distinct phonological inventories. Upon recognizing the predominantly sad or joyful content of a poem, readers are likely to adjust their prosody—including the prosody of silent reading (for a review of the role of phonology in silent reading, see Clifton, 2015)—to the content of the poems. Since several studies report that the vocalizations of joy and sadness have their own acoustic profiles (Scherer, 1986; Banse and Scherer, 1996; Paulmann, 2006), readers may end up perceiving their own inner prosody along the lines of the phenomenological distinction in question. This hypothesis was not tested in previous research. In the light of the fact that we could not confirm any of the hypotheses and results that we retested, the role of emotional prosody should be considered in future research on the topic.

In conclusion, our study confirms that the perception of tonal contrast (bright vs. dark) is dependent on the joyful or sad tonality of poems. However, it does not support the hypothesis that the frequencies of occurrence of particular phoneme classes predicts the perception of tonal contrast or the emotional classification of poems.

Therefore, a favorite idea of both philosophical speculation and linguistic accounts of poetry, while not being wholly discredited, still awaits a proper proof. Replication studies, while not a popular genre, are clearly important for scientific progress (Popper, 1959/2005). Ours amounts to the sober recognition that, at least for the time being, previous hypotheses of phono-emotional iconicity appear to be little more than “mimological reveries” (Genette, 1995, p. 210), however tempting such reveries about an inherent relation between sound material and emotional perception might be.

## Author contributions

MK and WM jointly designed the study, interpreted the data and wrote the paper. MK compiled the poetic corpus, gathered behavioral data, and conducted behavioral and phonological data analyses.

## Funding

Data acquisition for this paper was made possible through the support of the Research Cluster Languages of Emotion (EXC302), which was funded by the German Research Association DFG and hosted by the Freie Universität Berlin. The writing was conducted at the Max Planck Institute for Empirical Aesthetics in Frankfurt am Main, Germany.

### Conflict of interest statement

The authors declare that the research was conducted in the absence of any commercial or financial relationships that could be construed as a potential conflict of interest.

## Footnotes

^1^The perception of tonal contrasts is not considered to be limited to phenomena of synesthesia (Ramachandran and Hubbard, 2001; Ward and Mattingley, 2006; Simner, 2007; Cytowic and Eagleman, 2009; see Moos et al., 2014, for a comparative study of the tonal contrast perceptions of synaesthetes and non-synaesthetes in relation to a high second formant).

^2^Fechner's finding, however, was solely based on associations between tonal perception and graphemes and not on the underlying phonemes.

^3^Note that Tsur's focus lies not on the phonological material but rather on the acoustic articulation of poems of certain speakers and therefor on the phonetic but not phonological representation of sound.

^4^Referring in particular to Wiseman and Van Peer (2003) as well as to Fónagy (1961) and the contradictory results of these studies, Tsur (2012; see also Gafni and Tsur, 2015) offers an explanative interpretation from a perspective that takes into account the acoustic dimension of speech. In this opinion, “plosives are adequate for expressing both joy and aggressiveness, because the phonetic structure of plosives resembles the aesthetic structure of joyful and aggressive emotions” (Gafni and Tsur, 2015, p. 51), while nasals can express grief as well as tender moods. Since this interpretation, however, is not based on empirical data, the reported contradictions remain unresolved.

^5^Certainly, joy is not a dominant topic in poetry. Since most of our select poems were published in an acknowledged anthology (Reschke, 1992; cf. Gernhardt, 2012) we however ascribe a certain representative character to our sample of poems.

^6^Following the examples in Meier (1964), in the comparison we treated diphthongs as monophonemic (see also, e.g., Trubetzkoy, 1939) and affricates as biphonemic units (see also, e.g., Moulton, 1962; for a critical review of complex phonological segments, see Wiese, 1996, pp. 13–15).

^7^These differences were obtained by subtracting the values in Meier (1964) from the values for our corpus.

^8^Note that Wiese's matrix is based on a list of phonemes that is “maximal in the sense that every segment ever considered as a serious candidate for phonemic status has been included” (Wiese, 1996, p. 11). Further, since Jakobson (1962) and Tsur (1992) do not provide clear distinctions for vowel length and/or tenseness, we subsumed all phonemes that can be considered an /i/, /e/, /u/, or /o/ in Modern Standard German but differ in vowel quantity (i.e., /i:, ɪ, e:, ɛ, ɛ:, u:, ʊ, o:, ɔ/). We did not consider the vowel allocations of studies that define /a/ as a front but not a centralized vowel (e.g., Fechner, 1876; Moos et al., 2014).

^9^Given the hypotheses and research questions on which we focus in this study, some further items from the questionnaire were not considered in the analyses presented here.

^10^To be exact, Albers's corpus included 12 distinct poems and two different versions of Goethe's *Der König von Thule*.
